# *Bacteroides fragilis* participates in the therapeutic effect of methotrexate on arthritis through metabolite regulation

**DOI:** 10.3389/fmicb.2022.1015130

**Published:** 2022-12-15

**Authors:** Bailing Zhou, Chunyan Dong, Binyan Zhao, Ke Lin, Yaomei Tian, Rui Zhang, Lixin Zhu, Hueng Xu, Li Yang

**Affiliations:** ^1^State Key Laboratory of Biotherapy and Cancer Center, West China Hospital, Sichuan University and Collaborative Innovation Center of Biotherapy, Chengdu, Sichuan, China; ^2^Guangdong Institute of Gastroenterology, Guangdong Provincial Key Laboratory of Colorectal and Pelvic Floor Diseases, Department of Colorectal Surgery, The Sixth Affiliated Hospital, Sun Yat-sen University, Guangzhou, Guangdong, China

**Keywords:** methotrexate, rheumatoid arthritis, gut microbiota, *B. fragilis*, butyrate

## Abstract

Methotrexate (MTX) is a preferred disease-modifying anti-rheumatic drug in the management of rheumatoid arthritis (RA). However, the toxicity and inefficiency of MTX limit its clinical application. Gut microbiota has been implicated in the side effects and efficacy of MTX. In this study, the analysis of the gut microbiota in RA patients revealed that the abundances of intestinal *Bacteroides fragilis* was reduced after MTX treatment. We observed that MTX has no obvious therapeutic effect in the absence of *B. fragilis*, while transplantation of *B. fragilis* restored the efficacy of MTX in antibiotics-pretreated collagen-induced arthritis (CIA) mice. In addition, *B. fragilis* gavage was accompanied by an increase in butyrate. Supplementation of butyrate restored the response to MTX in gut microbiota-deficient mice, to a similar level achieved by *B. fragilis* gavage. These results show that gut microbiota-regulated butyrate plays an essential role in the efficacy of MTX, which will provide new strategies to improve the effectiveness of methotrexate in RA treatment.

## Introduction

Rheumatoid arthritis (RA) is a systemic autoimmune disease characterized by chronic synovial inflammation, cartilage and bone damage, and is associated with progressive disability, systemic complications as well as early death (Mcinnes and Schett, 2011). With an incidence of 0.5–1%, RA has been one of the most common chronic inflammatory diseases and adds a series of burdens to individuals, families and society (Smolen et al., 2016). Disease-modifying antirheumatic drugs (DMARDs) are widely used as first-line RA drugs due to their clinical efficacy and cost effectiveness. As one of DMARDs, methotrexate (MTX) has been commonly used in the treatment of RA since the 1980s (Weinblatt et al., 1985; Bedoui et al., 2019). Whereas, the clinical application of MTX is still limited by adverse events and unsatisfactory therapeutic effects. More than 75% of patients with low-dose MTX treatment suffer several common side effects, such as gastrointestinal toxicities, hepatotoxicity and so on (Iannone et al., 2016; Wang et al., 2018). In addition, about one-third of patients do not respond to MTX (Weinblatt et al., 1994; Saevarsdottir et al., 2011). Although several strategies have been used to manage side effects of MTX, such as the treatment with folic acid and changing the way of administration, the results are not ideal because of the decreased efficacy of MTX or other adverse effects (Tishler et al., 1988; Morgan et al., 1994; Wang et al., 2018). Therefore, there is still an urgent need to alleviate the side effects and to improve the efficacy of MTX.

The gut microbiota is closely related to the development of cancers and autoimmune diseases (Kamada et al., 2013; Garrett, 2015; Zitvogel et al., 2017; Yachida et al., 2019). In addition to this, there is accumulating evidence that the gut microbiota plays a crucial role in the toxicity and efficacy of various drugs (Kang et al., 2013; Alexander et al., 2017; Weersma et al., 2020). Studies demonstrate that cyclophosphamide causes changes in the composition of gut microbiota in mice and promote the transfer of some gram-negative bacteria to secondary lymphoid organs (Viaud et al., 2013). The bacteria stimulate a specific subset immune cells and enhanced immune responses. Moreover, cyclophosphamide has no effect on sterile mice, suggesting that the gut microbiota help define the anticancer effects of cyclophosphamide (Viaud et al., 2013). Similarly, two studies have shown that gut microbiota can modulate responses to PD-1-based immunotherapy in mice and in patients, confirming the importance of gut microbiota in the efficacy of drugs (Matson et al., 2018; Routy et al., 2018). In our previous study, we observed that MTX treatment led to alteration in the diversity and composition of the gut microbiota, with significantly decreased abundance in *Bacteroides fragilis* (*B. fragilis*), (Zhou et al., 2018) *which* is a prominent human commensal. It was shown that *B. fragilis* can inhibit T cell-mediated inflammation and prevent intestinal inflammatory diseases, such as colitis (Wexler and Goodman, 2017). The immunomodulatory molecule polysaccharide A (PSA), a component of the *B. fragilis*, induces an anti-inflammatory immune response mediated by IL-10 produced by T cells in intestinal tissue (Mazmanian et al., 2008; Round and Mazmanian, 2010). In addition to PSA, *B. fragilis* can regulate immune cells through short-chain fatty acids (SCFAs) (Su et al., 2020). Our previous data suggested that *B. fragilis* ameliorated MTX-induced mucositis by modulating macrophage polarization. Since the regulatory effect of *B. fragilis* in MTX-induced gastrointestinal toxicities has been known (Zhou et al., 2018), it is of interest to investigate the role of *B. fragilis* in the efficacy of MTX in RA.

In this study, we found that RA patients who were treated with MTX exhibited lower abundances of *B. fragilis*. Then we established a collagen-induced arthritis (CIA) model and utilized antibiotics to remove *B. fragilis* in mice. *B. fragilis*-deficient CIA mice were lack of response to MTX treatment. Meanwhile, supplementation with *B. fragilis* restored the efficacy of MTX in antibiotic-treated mice. We observed that *B. fragilis* stimulated the production of immunomodulatory M2 macrophages. In addition, we found that *B. fragilis* supplementation led to elevated production of butyrate and that butyrate restored the therapeutic effect of MTX in gut microbiota-deficient CIA mice. These data suggest that *B. fragilis* is critical for the therapeutic effect of MTX in RA.

## Materials and methods

### Patient fecal samples

Stool samples were collected from 21 RA patients with MTX treatment at day 0 and day 30 in the Affiliated Hospital of Zunyi Medical University. Patients were provided a feces collection tube to collect stool sample at home. The samples were sent to the lab within 24 h after collection. Stool bacterial DNA was isolated using the Stool DNA Isolation Kit (Foregene, Chengdu, China). The DNA and the rest of the sample was stored at −80°C. All human studies were approved by the Ethics Committee of Affiliated Hospital of Zunyi Medical University. Written informed consents were received from all patients prior to inclusion in the study.

### 16S rRNA amplicon sequencing and data analysis

The fecal samples were collected from CIA mice on day 0 and day 30 after MTX treatment. All samples were stored at –80°C. Stool bacterial DNA was extracted using the Stool DNA Isolation Kit (Foregene, Chengdu, China). One nanogram of purified fecal DNA was used for PCR amplification. Amplicons spanning the variable region 4 (V4) of the 16S rRNA gene were generated by using the following primers: forward, 5′-GTGCCAGCMGCCGCGGTAA-3′; reverse, 5′-GGACTACHVGGGTWTCTAAT-3′. The PCR products were then sequenced on an Illumina Hi-seq sequencer at Novogene (Novogene, Beijing, China). Paired-end reads from the original DNA fragments were merged by using FLASH (Magoc and Salzberg, 2011). Paired-end reads was assigned to each sample according to the unique barcodes. Sequences were analyzed using QIIME software package (quantitative insights into microbial ecology) (Caporaso et al., 2010). Sequences with ≥97% similarity were assigned to the same operational taxonomic units (OTUs). Taxonomical classification was performed using the RDP-classifier. The alpha diversity (such as ACE) for each subsample was calculated in Mothur. The unpaired, two-tailed t test was used to calculate differences between means (GraphPad Software). Principal component analysis (PCA) and principal coordinate analysis (PCoA) clustering were conducted using R. The linear discriminant analysis (LDA) with effect size (LEfSe) method of analysis was used to compare abundances of all bacterial clades using the Kruskal–Wallis test at a pre-defined α of 0.05. Significantly different taxa resulting from the comparisons of abundances between groups were used as input for LDA.

### Mice and generation of CIA model

Male 8-week-old DBA/1j mice were purchased from Beijing Vital River Laboratories Animal Technology Co. Ltd. All mice were maintained in a pathogen-free animal facility. All experimental procedures and animal care were approved by the Animal Care Committee of Sichuan University and were performed in accordance with the relevant ethical guidelines (Guidelines for Ethical Review of laboratory Animal Welfare No. GB/T 35892-2018). The method for the generation of CIA model was described previously (Zhou et al., 2019).

### Treatment of CIA mice

In this study, we performed four experiments to explore the relationship between gut microbiota and MTX efficacy in CIA mice. Mice in MTX-treated group were intraperitoneally (i.p.) injected with 1 mg/kg of MTX (Sigma-Aldrich, USA) every 3 days for 30 days. Control mice received PBS only. In the second and third experiments, 1mg/ml or a combination of antibiotics (Abs) (1 mg/ml ampicillin + 5 mg/ml streptomycin + 1 mg/ml metronidazole) were added in sterile drinking water of antibiotics-treated group on day −7 to day 0, The solutions and bottles were changed every 3 days. In Abs + MTX + *B. fragilis* and Abs + MTX + *Escherichia coli* group, mice were given oral gavage with 1 × 10^9^ bacterial cells on day 0. A total of 100 mM butyrate (But) (Sigma-Aldrich, USA) was dissolved in drinking water in Abs + MTX + But group on day 0 for 30 days. Each group consisted of 6 or 10 mice.

### Quantitative real-time PCR

Quantitative real-time PCR (qPCR) was conducted on Bio-rad CFX Connect platform using the SYBR Fast qPCR Mix (Takara, Japan) to detect the abundance of 16 rRNA gene in fecal bacterial DNA. Gene specific primer sequences were as follows: 16S rRNA (F: CGGTGAATACGTTCCCGG, R: TACGGCTACCTTGTTACGACTT), *B. fragilis* (F: TGATTC CGCATGGTTTCATT, R: CGACCCATAGAGCCTTCATC).

### Cultivation of bacteria

*Bacteroides fragilis* (ATCC 25285) was cultured on brain heart infusion (BHI) blood agar plates (Oxoid, USA) for 48 h at 37°C under anaerobic conditions. *B. fragilis* was harvested from the plates and suspended in sterile PBS. *E. coli* was cultured in Luria-Bertani liquid medium for 16 h at 37°C before harvest. Then, the bacteria were washed with PBS and resuspended in sterile PBS to achieve an OD_600_ = 1, which corresponds to approximately 1 × 10^9^ colony forming units (CFUs) per ml.

### Flow cytometry

Spleens were harvested from mice on day 30 after the first injection of MTX. The tissues were cut into small pieces and filtered through a 70 μm cell strainer. The cells were stained with antibodies against the following surface markers: PerCP-CD11b, PE-F4/80 and FITC-CD206. These antibodies were purchased from BD Biosciences. Cell detection were conducted on a flow cytometer (FACSCalibur and Accuri C6, BD Biosciences), the data were analyzed with FlowJo 6.0 and NovoExpress.

### Radiological and histological assessment of joint tissues

On day 30, a micro-CT (PerkinElmer, USA) was used to assess the degree of joint injury. Then, mice were sacrificed and joint tissues were harvested. After fixation in 4% paraformaldehyde for 24 h, the tissues were decalcified with EDTA and embedded in paraffin. Finally, 4 μm sections of the joint tissues were prepared and stained with HE.

### Metabolomics analysis based on GC-MS

Fecal samples were collected on day 7 after MTX treatment with or without *B. fragilis* gavage. 50 μl 15% phosphoric acid, 100 μl 125 μg/mL isohexanoic acid solution and 400 μl diethyl ether was added to 50 mg feces sample. After homogenate for 1min, the mixture was centrifuged at 12,000 RPM at 4°C for 10 min, and the supernatant was used for short-chain fatty acids (SCFAs) analysis by GC-MS.

### Butyrate treatment *in vitro*

Cells isolated from the spleen were stimulated with lipopolysaccharide (LPS, 100 ng/ml) with or without butyrate (100 μM–2 mM) for 24 h, after which cells were collected for flow cytometry.

### Statistical analysis

Data were analyzed using GraphPad Prism 8 (GraphPad, La Jolla, CA, USA). Data were depicted as the means ± SEM, and statistical comparisons were conducted using t-test or unpaired one-way analysis of variance (ANOVA). *p* < 0.05 was considered statistically significant.

## Results

### MTX therapy alters the composition of gut microbiota in RA patients

**FIGURE 1:**
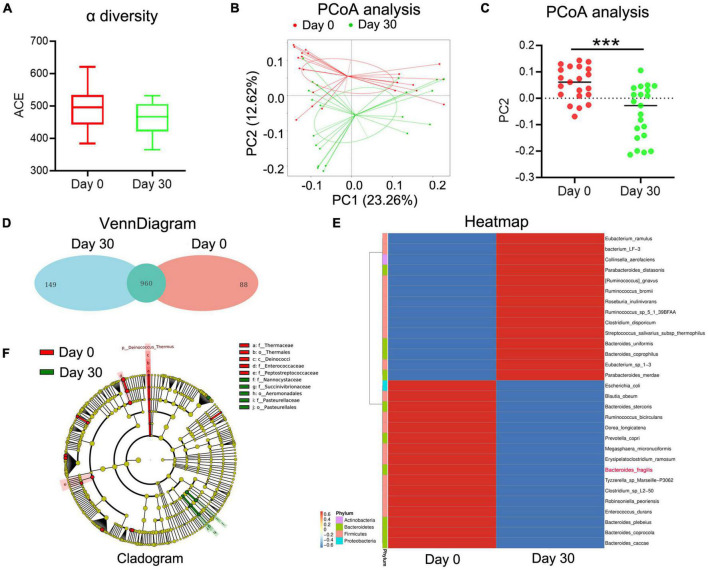
The changes of gut microbiota in MTX-treatment patients. Stool samples were collected from 21 RA patients with MTX treatment at day 0 and day 30. Stool bacterial DNA was extracted using the Stool DNA Isolation Kit and detected using 16S rRNA amplicon sequencing. **(A)** ACE analysis to predict species richness among different groups. **(B,C)** PCoA to assess differences between microbial communities. **(D)** Flower figure of VennDiagram to show the common and unique OTUs among different groups. **(E)** Heatmap to present the change of different species. **(F)** Cladogram to analyze different species between groups. ***p < 0.001.

In our previous study, we observed that the abundance of *B. fragilis* was decreased in normal *Balb/c* mice after MTX treatment. To evaluate whether MTX influences the composition of intestinal microbiota in arthritis, stool samples were collected from 21 RA patients before and after MTX treatment. 16S rRNA gene amplicon sequencing was conducted to assess the relationship between gut microbiota and clinical response. Unlike the results of animal experiments, the alpha diversity of gut bacteria did not change significantly after MTX treatment in all patients (Figure 1A). PCoA and venn diagram showed that the composition of gut microbiota was altered after MTX treatment (Figures 1B–D). Clustering heatmap of species abundance showed the changes of 30 bacteria, among which 14 bacteria had increased, such as *Eubacterium ramulus*, and 16 bacteria had decreased, such as *Escherichia coli* (Figure 1E). Notably, the content of *B. fragilis* was reduced (Figure 1E), which is in agreement with the results in our previous study, confirming that *B. fragilis* is one of gut microbiota that related to the MTX treatment.

### Transplantation of *B. fragilis* enhances the effect of MTX in gut microbiota-deficient mice

**FIGURE 2:**
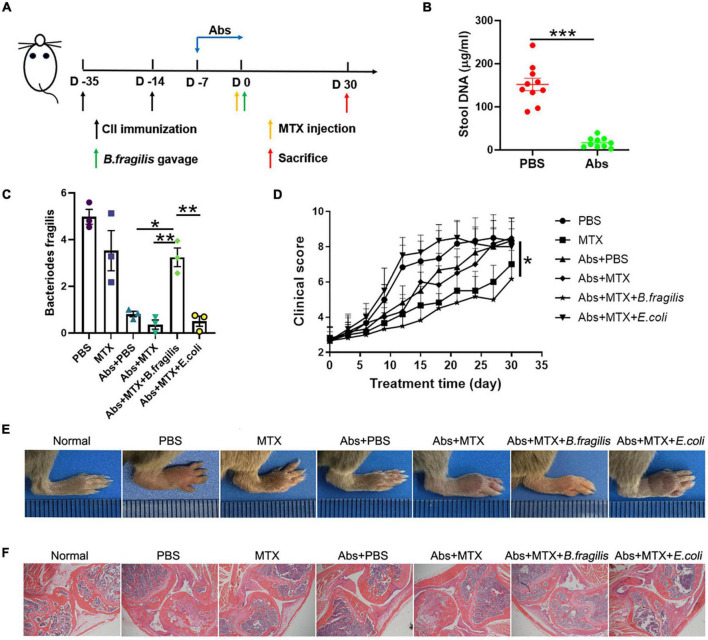
The response to MTX was affected by *Bacteroides fragilis* in gut microbiota deficient CIA mice. **(A)** Schematic of treatments. Mice were treated with antibiotics at the onset of arthritis. Mice received *B. fragilis* gavage and MTX injection on day 0. **(B)** The total stool DNA after Abs treatment (*n* = 10). **(C)** The content of *B. fragilis* after gavage (*n* = 3). **(D)** Clinical scores of CIA mice (*n* = 6). **(E)** The photos of hindlimb. **(F)** HE staining of joint tissues (magnification is 40×). Data are presented as the mean ± SEM. *p < 0.05, **p < 0.01, ***p < 0.001. Abs, antibiotics.

To further verity the role of *B. fragilis* in the treatment of RA with MTX, the CIA mice were pre-treated (or untreated as controls) with antibiotics for 7 days, followed by MTX injection with or without bacteria (*B. fragilis* or *E. coli*) transplantation (Figure 2A). The concentration of stool DNA in the mice treated with antibiotics was significantly lower than that of the mice untreated with antibiotics (Figure 2B), indicating that the gut microbiota was effectively reduced. After bacteria gavage, transplantation with *B. fragilis* was able to restore the abundance of *B. fragilis* (Figure 2C). Consistent with the above experiment, the disease severity evaluation with the clinical score data showed that MTX treatment could not achieve a satisfactory result in the gut microbiota-deficient mice (Figure 2D). Compared to the MTX group, the arthritis degree of the mice in Abs + MTX group was not alleviated effectively (Figure 2D). However, the inhibitory effect of MTX on arthritis in antibiotics-treated mice was enhanced after *B. fragilis* gavage, indicated by the delayed development of CIA in the Abs + MTX + *B. fragilis* group, comparable to that in the MTX group (Figure 2D). *E. coli* gavage did not yield a similar effect as *B. fragilis*, no difference in clinical score was observed between the PBS and the Abs + MTX + *E. coli* group (Figure 2D). In addition, the swelling of joints was not evident in the MTX or the Abs + MTX + *B. fragilis* group on day 30 (Figure 2E). HE staining showed that synovial hyperplasia, cartilage injury, lymphocyte infiltration and bone erosion of the knee joint in CIA mice were relieved in the MTX and the Abs + MTX + *B. fragilis* groups (Figure 2F). It appears that *B. fragilis* could restore the therapeutic effect of MTX in gut microbiota-deficient mice.

**FIGURE 3:**
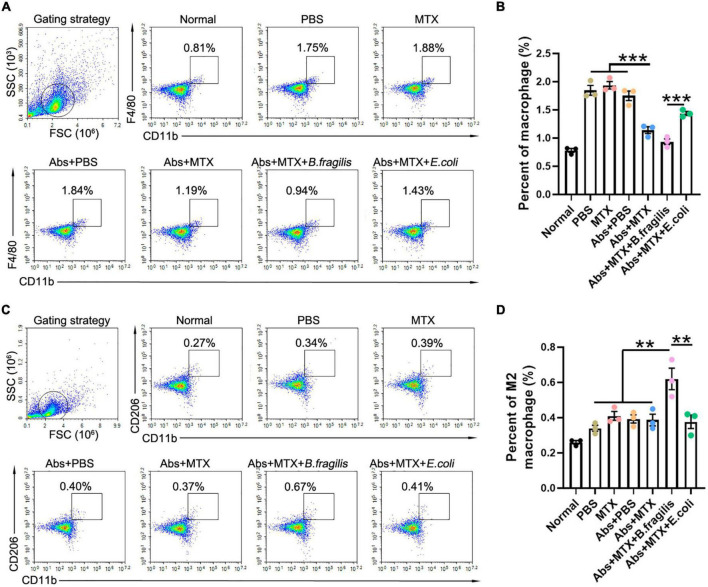
*Bacteroides fragilis* alters the polarization of macrophage. **(A–D)** FACS analysis of splenic cells. Percentages of **(A,B)** macrophages and **(C,D)** M2 macrophages (*n* = 3). Data are presented as the mean ± SEM. **p < 0.01, ***p < 0.001. Abs, antibiotics.

Our previous data suggested that *B. fragilis* treatment can lead to significant alternation in macrophages, which also play a critical role in RA (Quero et al., 2017). Therefore, we investigated the inflammatory status of spleen in mice. MTX and antibiotic treatment did not alter the amount of splenic CD4^+^F4/80^+^ macrophage and CD11b^+^CD206^+^ M2 macrophage in CIA mice, while there was a prominent up-regulation of M2 macrophage in the Abs + MTX + *B. fragilis* group, confirming the role of *B. fragilis* in regulating immune cells in MTX-treated CIA mice (Figures 3A–D).

### *B. fragilis* gavage promotes butyrate metabolism in methotrexate-treated mice

**FIGURE 4:**
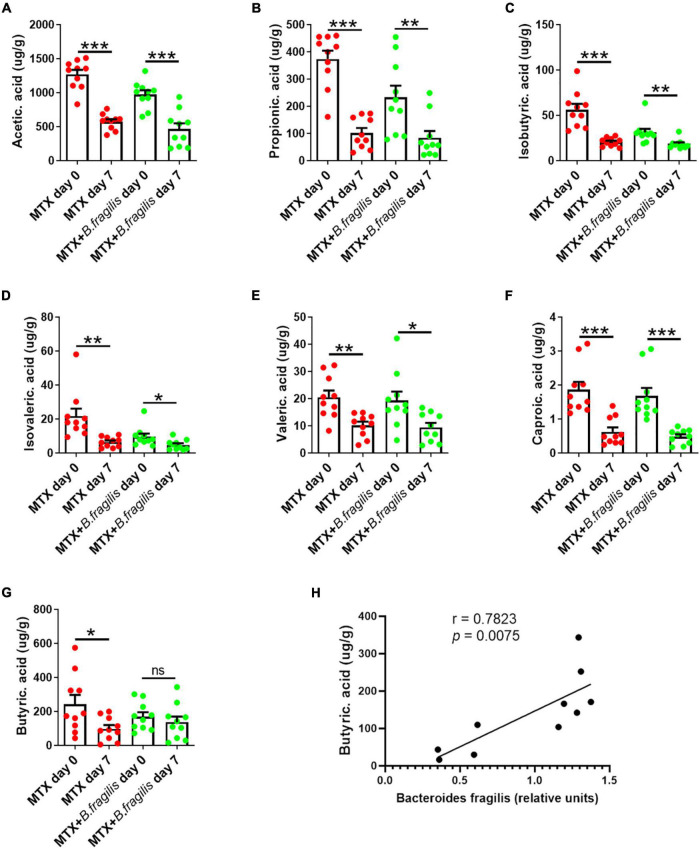
Analysis of SCFAs in feces of mice. MTX-treated CIA mice were gavaged with PBS or PBS containing *B. fragilis* for 7 days. Fecal samples were collected for GC-MS analysis of **(A)** acetic acid, **(B)** propionic acid, **(C)** isobutyric acid, **(D)** isovaleric acid, **(E)** valeric acid, **(F)** caproic acid and **(G)** butyric acid (butyrate), (*n* = 10). **(H)** Correlation analysis between and the relative units of *B. fragilis* and the content of butyrate. Data are presented as the mean ± SEM. *p < 0.05, **p < 0.01, ***p < 0.001.

*Bacteroides* produces SCFAs in intestine (Macfarlane and Macfarlane, 2003). One relevant question is whether *B. fragilis* gavage affects the production of SCFAs in MTX-treated mice. Therefore, the feces of MTX-treated mice with/without *B. fragilis* gavage were harvested for SCFAs analysis. We found that the concentration of acetic acid, propionic acid, isobutyric acid, butyric acid (butyrate), isovaleric acid and valeric acid was markedly reduced after MTX administration (Figures 4A–G). In addition to butyrate, other SCFAs also decreased in mice of MTX + *B. fragilis* group (Figures 4A–G), suggesting *B. fragilis* gavage promoted the production of butyrate. To verify the correlation between the content of *B. fragilis* and the production butyrate, the relative units of *B. fragilis* was detected. Correlation analysis showed that the person r = 0.7823 (*p* < 0.01) (Figure 4H), indicating a positive correlation between content of *B. fragilis* and butyrate. These data reveal that *B. fragilis* gavage could enhance the metabolism of butyrate in MTX-treated mice.

### Supplementation of butyrate recovers the effect of MTX in gut microbiota-deficient mice

**FIGURE 5:**
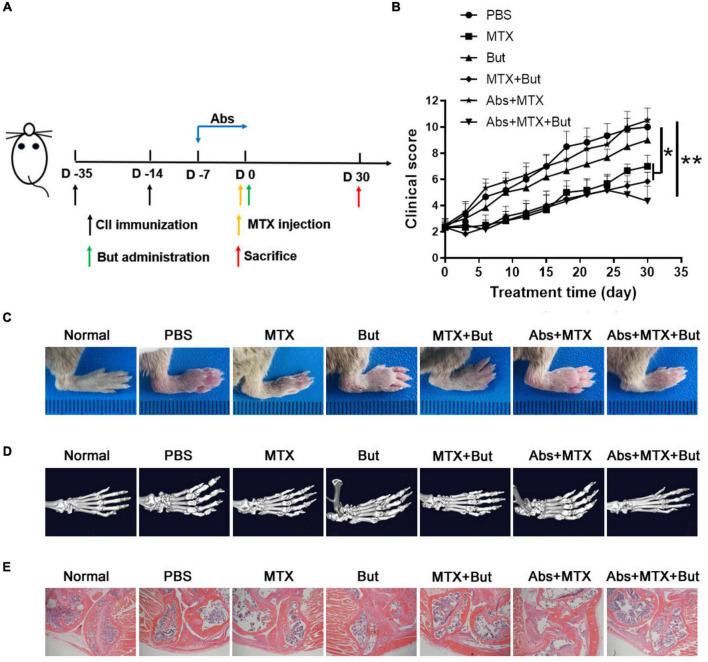
Butyrate improves the efficacy of MTX in gut microbiota-deficient mice. **(A)** Schematic of treatments. Mice were treated with antibiotics at the onset of arthritis. Mice received butyrate administration and MTX injection on day 0. **(B)** Clinical scores of CIA mice (n = 6). **(C)** The photos of hindlimb. **(D)** Micro-CT assessment of paws. **(E)** HE staining of joint tissues (magnification is 40×). Data are presented as the mean ± SEM. *p < 0.05, **p < 0.01. Abs, antibiotics; But, butyrate.

As butyrate is mainly produced by the gut microbiota (Flint et al., 2015; Forslund et al., 2015), and is an important regulator of immune response (Liu et al., 2018; Silva et al., 2018), we sought to determine the influence of butyrate administration on the MTX treatment in CIA mice with/without pretreatment of antibiotics. Butyrate was provided in drinking water until the end of the experiment (Figure 5A). There was no obvious remission of the arthritis score in CIA mice with butyrate treatment alone (Figure 5B). Moreover, butyrate did not affect the efficacy of MTX in CIA mice untreated with antibiotics (Figure 5B). Nevertheless, the development of arthritis was effectively inhibited in the Abs + MTX + But group compared to that in the Abs + MTX group (Figure 5B). Consistently, butyrate improved the swelling, deformation, cartilage injury, lymphocyte infiltration and bone erosion of joints in the Abs + MTX + But group (Figures 5C–E). Taken together, butyrate can restore the efficacy of MTX in gut microbiota-deficient mice as well as *B. fragilis*.

**FIGURE 6:**
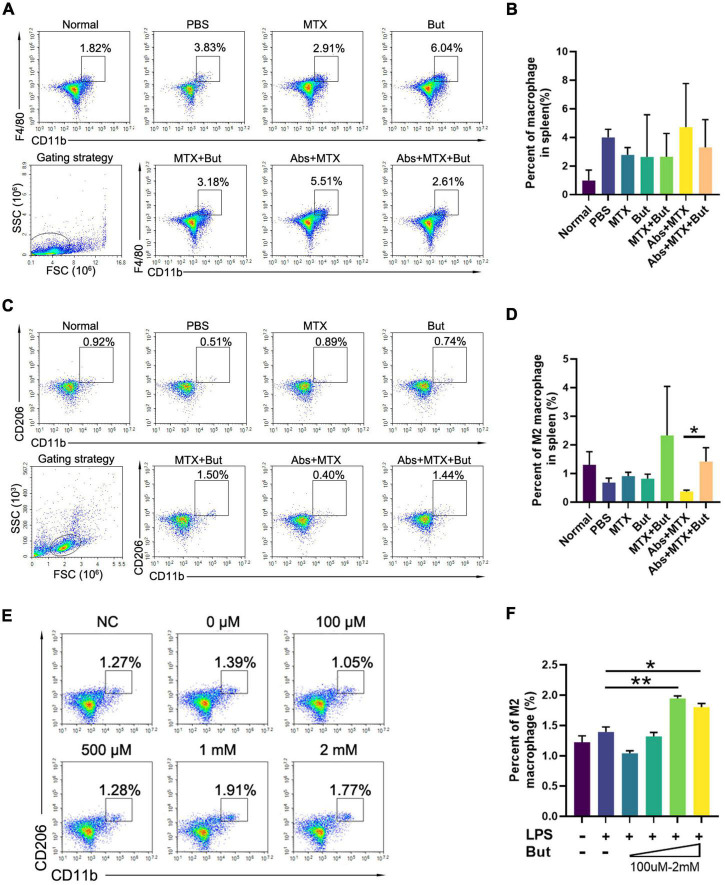
The impact of butyrate on macrophage. **(A–D)** FACS analysis of splenic cells. **(A,B)** Macrophages and C,D M2 macrophages (*n* = 3). **(E,F)** FACS analysis of macrophage *in vitro*. Cells isolated from the spleen were stimulated with lipopolysaccharide (LPS, 100 ng/ml) with or without butyrate (But, 100 μM–2 mM) for 24 h before FACS analysis. The alteration of **(E,F)** M2 macrophages (*n* = 3) were recorded. Data are presented as the mean ± SEM. *p < 0.05, **p < 0.01. Abs, antibiotics; But, butyrate.

We next investigated the impact of butyrate on splenic macrophage. Butyrate promoted the expansion of M2 macrophage in CIA mice with MTX injection (Figures 6A–D). In order to test whether butyrate exerts the same effect on lymphocytes *in vitro*, splenic lymphocytes were isolated from CIA mice and stimulated with LPS and butyrate. We found that the percent of M2 macrophage was increased in the presence of butyrate in a dose dependent manner (Figures 6E, F). These data indicated that butyrate can regulate the immune response by promoting the development M2 macrophage both *in vivo* and *in vitro*.

## Discussion

Gut toxicity and lack of efficacy are still challenging problems in the clinical treatment of RA with MTX. MTX is an antagonist of folate, the folate metabolism pathway also exists in microbiota, the intestinal microbial compositions likely change after MTX treatment (Huang et al., 2020). In addition, MTX-induced mucositis can also lead to bacterial translocation (Ubeda et al., 2010). Previous studies showed that gut microbiota can relieve the toxicity of various drugs, including MTX (Zhou et al., 2018; Huang et al., 2020). Here, with CIA models, we verified the role of gut microbiota in the efficacy of MTX in RA, and further demonstrated that butyrate-regulating *B. fragilis* is an intestinal bacteria related to the efficacy of MTX.

Studies have reported that *B. fragilis* can alleviate MTX-induced intestinal inflammation (Zhou et al., 2018). Since gut microbiota has been proven not only to reduce the intestinal toxicity of drugs, but also to affect their efficacy (Alexander et al., 2017; Weersma et al., 2020), and the detection of gut microbiota of RA patients showed the content of *B. fragilis* was decreased after MTX therapy, we speculated that transplantation of *B. fragilis* may enhance the therapeutic effect of MTX in gut-deficient CIA mice. To test our hypothesis, we conducted *B. fragilis* gavage in MTX-treated mice after removing microbiota with antibiotics. While the arthritis-inhibitory effect of MTX was lowered after the clearance of gut microbiota, supplementation with *B. fragilis* could restore the therapeutic effect to a similar level found in antibiotics-untreated mice, which did not occur with the supplementation of *E. coli*. Anti-inflammatory M2 macrophages are crucial in the pathogenesis of immune-inflammatory disorders (Tardito et al., 2019). Our observation that *B. fragilis* up-regulated the number of M2 macrophage suggested that *B. fragilis* contributed to the therapeutic effects of MTX by regulating the development of anti-inflammatory lymphocytes.

The mechanisms of intestinal microbiota affecting drug efficiency include metabolism, immune regulation, translocation, enzymatic degradation and ecological variation (Panebianco et al., 2018). Gut microbiota metabolizes complex dietary carbohydrates through a large number of enzymes, and degrades dietary fiber to produce organic acids, gases and a large amount of SCFAs (Martin-Gallausiaux et al., 2021). Gut microbiota regulate the function of immune cells through its metabolites SCFAs (Rooks and Garrett, 2016; Ratajczak et al., 2019). These biological functions of SCFAs are mediated by SCFA receptors GPR41 and GPR43, expressed on immune cells, adipocytes, and intestinal cells (Tan et al., 2014; Sun et al., 2017). We found that the supplementation of *B. fragilis* prevented the MTX-induced decrease of butyrate, which can limits the autoimmune response (Takahashi et al., 2020). It is possible that butyrate is involved in the beneficial effect of *B. fragilis* on the efficacy of MTX. By adding butyrate to the drinking water during MTX treatment, we observed that butyrate restored the therapeutic effect of MTX in gut microbiota-deficient CIA mice, to a similar level achieved with *B. fragilis* gavage. Our *in vivo* and *in vitro* experiments confirmed that butyrate promote the proliferation of M2 macrophage. Macrophage polarization is a complex process of multi-factor interaction, which is regulated by a variety of intracellular signaling molecules and their pathways, including JAK/STAT signaling pathway, PI3K/Akt signaling pathway (Vergadi et al., 2017). Butyrate has been reported to improve inflammation by regulating the signaling pathway PI3K/Akt via GPRs (Pirozzi et al., 2018). Taken together, regulating the host immunity through the alternation in butyrate metabolism is one of the potential ways that that *B. fragilis* plays a role in MTX therapy. Notably, there is no evidence that *B. fragilis* is a producer of butyrate. Considering microbiota transplantation may alter the intestinal microenvironment and nutrient competition, thereby altering the gut microbiota population (Gu et al., 2016), we speculated that *B. fragilis* supplementation resulted in structural changes in gut microbiota and promoted the proliferation of butyrate-produced species, suggesting that more attention should be paid to the dynamics of intestinal microbiome after microbiota transplantation.

Although numerous studies have reported that metabolites of gut microbiota are associated with the progression of arthritis and suggest butyrate as a therapeutic strategy for arthritis, these studies have not specifically explored the relationship between gut microbiota produced-derived metabolites and drug therapy (Kim et al., 2018; Rosser et al., 2020; He et al., 2022; Martinsson et al., 2022). Based on the correlation between intestinal microbiota and drug toxicity, this study evaluated the influence of gut microbiota on drug efficacy, which is helpful to solve the problems of intestinal toxicity and unsatisfactory response of MTX. However, the comparison of 16S rRNA amplicon sequencing analysis results between the animal model and the patient showed that the changes of intestinal microbiota after MTX treatment were not completely similar, suggesting that the animal model could not completely represent the clinical influence of gut microbiota. We need to design reasonable clinical experiments to verify the role of *B. fragilis*. In addition, gut microbiota is a complex system that interacts in human to affect immune response. We have also observed changes in other bacteria in MTX treatment, which suggested other bacteria may also be involved in this effect except *B. fragilis*. Among these altered strains, *Escherichia coli* has been reported to suppress the development of arthritis in germ-free rats (Kohashi et al., 1986), but its effect on the MTX is not clear. In this study, we found that E transplantation had no significant effect on the therapeutic efficacy of MTX (Figures 2, 3). *Bacteroides uniformis* and *Prevotella copri* have also been shown to be associated with the development of arthritis (Miller et al., 2015; Seifert et al., 2022). The effect of these bacteria on MTX is also worth further investigation.

In summary, we found that MTX treatment was correlated to the abundances of *B. fragilis* in the gut of the RA patients. Next, we demonstrated that *B. fragilis* plays an essential role in the efficacy of MTX by regulating the metabolism of butyrate. These findings advocate for a potential microbial intervention strategy for improving the efficacy of MTX in RA management.

## Data availability statement

The data presented in the study are deposited in the jianguoyun repository (https://www.jianguoyun.com/p/DZIBy9MQzYaeCxj66OoEIAA).

## Ethics statement

The studies involving human participants were reviewed and approved by the Ethics Committee of Affiliated Hospital of Zunyi Medical University. The patients/participants provided their written informed consent to participate in this study. The animal study was reviewed and approved by Animal Care Committee of Sichuan University.

## Author contributions

LY contributed to study design. BLZ, CD, BYZ, KL, and HX contributed to data and figures collection. LZ contributed to writing. YT and RZ contributed to literature search. All authors contributed to the article and approved the submitted version.
